# Efficacy and safety of Ginkgolide with intravenous alteplase thrombolysis in acute ischemic stroke with large vessel occlusion: a subgroup analysis of GIANT

**DOI:** 10.3389/fphar.2024.1452174

**Published:** 2024-08-30

**Authors:** Zheyu Zhang, Wansi Zhong, Xuting Zhang, Xiaodong Ma, Xudong Lu, Meixia Zhang, Anyang Tao, Bing Zhang, Min Lou

**Affiliations:** ^1^ Department of Neurology, School of Medicine, The Second Affiliated Hospital of Zhejiang University, Hangzhou, China; ^2^ Department of Neurology, Haiyan People’s Hospital, Jiaxing, China; ^3^ Department of Neurology, The Second Affiliated Hospital of Jiaxing University, Jiaxing, China; ^4^ Department of Neurology, Affiliated Jinhua Hospital, Zhejiang University School of Medicine, Jinhua, China; ^5^ Department of Neurology, The First People’s Hospital of Taizhou, Taizhou, China; ^6^ Department of Neurology, Huzhou Central Hospital, Huzhou, China

**Keywords:** Ginkgolide, acute ischemic stroke, intravenous alteplase, minor stroke, large vessel occlusion

## Abstract

**Aim:**

This study aims to explore the effectiveness and safety of Ginkgolide^®^ in acute ischemic stroke (AIS) patients with large vessel occlusion (LVO) and moderate-to-severe stroke receiving intravenous alteplase thrombolysis (IVT).

**Methods:**

Ginkgolide with Intravenous Alteplase Thrombolysis in Acute Ischemic Stroke Improving Neurological Function (GIANT) was an open-label, prospective, multicenter, cluster-randomized clinical trial and included AIS patients in 24 centers randomized to the intervention of intravenous Ginkgolide^®^ or control group within the first 24 h after IVT. LVO was defined as any occlusion of the internal carotid artery, M1 or M2 of the middle cerebral artery, A1 or A2 of the anterior cerebral artery, P1 of the posterior cerebral artery, and V4 of the vertebral artery or the basilar artery. Stroke severity was assessed with the National Institutes of Health Stroke Scale (minor ≤5; moderate-to-severe >5). The primary outcome was a good outcome, defined as a modified Rankin Scale (mRS) score of 0–2 at 90 days. Secondary outcomes were early neurological improvement (ENI), defined as ≥18% increase in the National Institutes of Health Stroke Scale (NIHSS) score at 7 days compared to baseline and distribution of mRS at 3 months.

**Results:**

A total of 1,113 patients were included, with 268/913 (29.4%) presenting LVO and 508 (45.6%) presenting moderate-to-severe stroke. In patients with LVO, Ginkgolide^®^ usage was independently associated with ENI (*P* = 0.001) but not with a good outcome (*P* = 0.154). In the moderate-to-severe stroke subgroup, Ginkgolide^®^ was independently associated with both a good outcome (*P* = 0.009) and ENI (*P* = 0.028). Ginkgolide^®^ did not increase the risk of hemorrhagic transformation (all *P* > 0.05).

**Conclusion:**

Using Ginkgolide^®^ within 24-h after intravenous rt-PA is effective and safe in LVO and moderate-to-severe stroke patients.

## Introduction

Intravenous thrombolysis (IVT) is the only approved systemic reperfusion treatment for patients with acute ischemic stroke (AIS), according to the current guidelines (Powers et al., 2019; Yang et al., 2023). However, a large proportion of patients with a large vessel occlusion (LVO) and severe symptoms still had poor outcomes after IVT. In patients with LVO, up to 40% patients did not attain a good outcome despite successful recanalization (Li et al., 2023). In patients with severe anterior circulation stroke, this figure may reach 56.8% (González et al., 2013; Sarraj et al., 2021). Therefore, it is urgent to develop other treatment approaches combined with IVT to improve the clinical outcome in patients with LVO and severe symptoms.

LVO often presents with heavy clot burden, the formation of which involves the conversion of soluble fibrinogen to insoluble fibrin (Pikija et al., 2016; Chiang et al., 2022) and activation of arachidonic acid in increasing the clot length and diminishing clot lysis (Jones et al., 2022). A network pharmacology analysis showed that the fibrin clot formation pathway and arachidonic acid metabolism signaling pathways were significantly overlapped in AIS and ginkgolide injection (diterpene ginkgolide meglumine injection, comprising mainly ginkgolide), indicating that ginkgolide injection may relieve clot burden through the aforementioned pathways (Han et al., 2023). We thus assume that Ginkgolide^®^ may have additional benefits for LVO patients than for non-LVO patients. Our previously published Ginkgolide with Intravenous Alteplase Thrombolysis in Acute Ischemic Stroke Improving Neurological Function (GIANT) study has already proven that using Ginkgolide^®^ within 24 h after intravenous rt-PA was effective and safe (Zhang et al., 2021). In this study, we aimed to further explore the effectiveness and safety of Ginkgolide^®^ in patients with LVO and severe symptoms after IVT.

## Methods

### Study design and populations

GIANT was an open-label, prospective, multicenter, cluster randomized clinical trial involving 24 centers in China (NCT03772847). We enrolled patients who 1) were 18 years or older and 2) were AIS patients who met the criteria of IVT (Scheitz et al., 2017). We excluded patients who 1) were diagnosed as cerebral arteritis; 2) had baseline alanine aminotransferase or aspartate aminotransferase ≥3 times the upper limit of normal, or baseline serum creatinine ≥1.5 times the upper limit of normal; 3) were allergic to ginkgo drugs, alcohol, or glycerol; and 4) participated in other clinical trials. The criteria for intravenous thrombolysis were according to the current guideline (Powers et al., 2019).

Written informed consent was obtained from all patients or their legal representatives. The human ethics committee of the Second Affiliated Hospital of Zhejiang University (SAHZU), School of Medicine, approved the trial. The clinical trial was conducted according to the principle expressed in the Declaration of Helsinki.

### Data collection

The following patient characteristics were recorded from the registry database: demographics, risk factors (hypertension, diabetes, smoking history, stroke history, coronary heart disease (CHD), and atrial fibrillation (AF)), door to needle time (DNT), and the National Institutes of Health Stroke Scale (NIHSS) score at baseline, 24 h, and 7 days. Stroke subtypes, classified as large-artery atherosclerosis (LAA), small-artery occlusion (SAO), cardiac embolism (CE), undetermined etiology (UE), and other determined etiology (OE), were determined according to the Trial of Org 10172 in Acute Stroke Treatment (Adams et al., 1993). Data on all included patients were consecutively recorded in a secure, purpose-built, web-based data entry system.

LVO was defined as any occlusion of the internal carotid artery (ICA), A1 or A2 segment of the anterior cerebral artery (ACA-A1/A2), M1 or M2 segment of the middle cerebral artery (MCA-M1/M2), P1 segment of the posterior cerebral artery (PCA-P1), V4 segment of the vertebral artery (VA-V4), or basilar artery (BA) based on magnetic resonance angiography, computed tomographic angiography, or digital subtraction angiography. Moderate-to-severe stroke was defined as an admission NIHSS score >5, and minor stroke was defined as an admission NIHSS score ≤5 (de Jong et al., 2003; Xiao and Harrison, 2020).

### Outcome assessment

The modified Rankin Scale (mRS) score at 90 days of all the included patients was followed up with telephone questionnaires by external clinical evaluators who were blinded to the patients’ clinical data. The primary outcome was a good outcome, defined as mRS 0–2 at 90 days. Secondary outcomes included early neurological improvement (ENI) defined as (baseline NIHSS - NIHSS at 7 days)/baseline NIHSS*100% ≥ 18% at 7 days (Zhang et al., 2021) and the distribution of mRS scores. The safety outcomes included any intracranial hemorrhage transformation (HT) and symptomatic intracranial hemorrhage (sICH) on the 7-day follow-up. HT was classified into hemorrhagic infarction (HI) and parenchymal hemorrhage (PH). An intracerebral hemorrhage that caused an increase of NIHSS ≥4 points was defined as symptomatic intracranial hemorrhage (sICH) (Koton et al., 2022). A computerized tomography (CT) scan was performed within 24 h and on 7 ± 1 day after thrombolysis for the assessment of hemorrhage. Additional images might be performed in the case of clinical worsening or at the discretion of the treating physicians.

### Statistical analysis

All numeric variables are expressed as mean ± standard deviation (SD) or median [interquartile range (IQR)]. Categorical variables are presented as frequencies (percentages). Chi-squared tests or Fisher’s exact tests were used to compare dichotomous variables between groups, while the Mann–Whitney U test was used for ordered categorical variables. The independent-sample two-tailed t-test or Mann–Whitney U test was used for continuous variables depending on the normality of the distribution. Associations of Ginkgolide^®^ usage with primary and secondary outcomes were determined by binary logistic regression models, respectively, after adjustment by baseline characteristics with *P* < 0.05 in univariate analyses. Multivariable ordinal logistic regression analysis was used to calculate the adjusted common odds ratio on the 90-day mRS score. All statistical analyses were conducted using SPSS (version 22.0; IBM Corp., Armonk, NY, United States). *P* < 0.05 was considered statistically significant.

## Results

### Overall characteristics

**FIGURE 1:**
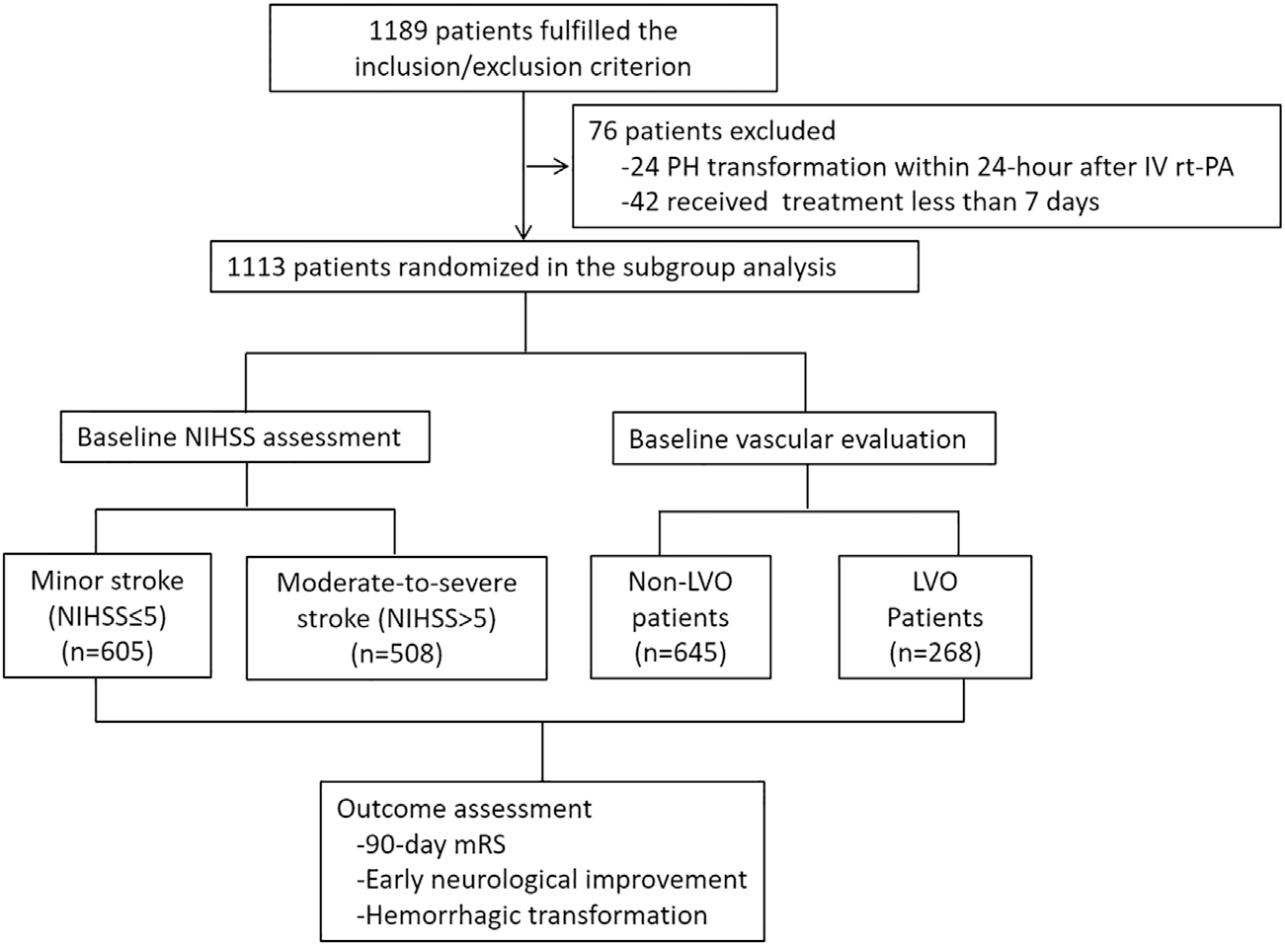
Patient recruitment flowchart.

From May 2018 to December 2019, a total of 24 hospitals and 1,113 patients were included in the subgroup analysis (Figure 1). Among them, 513 patients were in the Ginkgolide^®^ group, and 600 patients were in the control group. There were 508 (45.6%) minor stroke and 605 (54.4%) moderate-to-severe stroke patients. A total of 913 patients received vascular examination upon admission, and 268 (29.4%) patients were presented with LVO. A total of 63 patients were lost to follow-up, and 758/1,050 (72.2%) patients had a good outcome. With regard to safety outcome, 844 patients received 7-day CT scan, and 66 (7.8%) and 12 (1.4%) patients had HT and sICH, respectively.

Univariate analyses are shown in Supplementary Table S1. Compared with the control group, the Ginkgolide^®^ group was younger and had lower baseline NIHSS and DNT; a lower proportion of AF, LVO, and thrombectomy; and a higher proportion of smoking, hypertension, previous stroke, ENI (67.7% vs. 74.0%, *P* = 0.024), and 90-day good outcome (78.6% vs. 66.5%, *P* < 0.001). There were no significant differences in other variables.

### Comparison between LVO and non-LVO patients

Table 1 summarizes baseline characteristics in LVO and non-LVO patients. Compared with the non-LVO group, patients with LVO were older; had higher baseline NIHSS and DNT and 90-day mRS score; higher proportion of AF, HT, and sICH; and lower proportion of hypertension, diabetes, and Ginkgolide^®^ usage (38.1% vs. 46.7%, *P* = 0.017). There were no significant differences in other variables.

**TABLE 1:** Comparison between patients with minor stroke and moderate-to-severe stroke.

	Minor stroke (n = 605)	Moderate-to-severe stroke (n = 508)	*P*-value	Non-LVO (n = 645)	LVO (n = 268)	*P*-value
Age (year, IQR)	67 (58–74)	72 (62–81)	<0.001	67 (59–76)	70 (61–80)	0.013
Female (n, %)	225 (37.2)	227 (44.7)	0.011	267 (41.4)	106 (39.6)	0.606
Baseline NIHSS (IQR)	3 (2–4)	11 (8–16)	<0.001	4 (2–7)	12 (5–17)	<0.001
DNT (min, IQR)	44 (35–58)	47 (38–62)	0.003	44 (35–58)	48 (38–64)	0.003
Ginkgolide^®^ usage (n, %)	302 (49.9)	211 (41.5)	0.005	301 (46.7)	102 (38.1)	0.017
Risk factors (n, %)						
Smoking	212 (35.0)	161 (31.7)	0.238	216 (33.5)	93 (34.7)	0.724
Hypertension	396 (65.5)	320 (63.0)	0.393	428 (66.4)	153 (57.1)	0.008
Atrial fibrillation	78 (12.9)	131 (25.8)	<0.001	89 (13.8)	83 (31.0)	<0.001
Diabetes	112 (18.5)	62 (12.2)	0.004	119 (18.4)	17 (6.3)	<0.001
Coronary heart disease	30 (5.0)	66 (13.0)	<0.001	46 (7.1)	27 (10.1)	0.135
Previous stroke	72 (12.0)	64 (12.7)	0.727	75 (11.7)	30 (11.2)	0.836
Large-vessel occlusion (n, %)	68 (13.8)	200 (47.5)	<0.001	—	—	—
Thrombectomy (n, %)	—	—	—	—	35 (24.6)	0.061
ENI (n, %)	410 (68.2)	367 (73.4)	0.060	479 (74.4)	170 (64.2)	0.002
Follow-up HT (n, %)	9 (2.0)	57 (14.5)	<0.001	13 (2.7)	48 (21.8)	<0.001
Follow-up sICH (n, %)	2 (0.4)	10 (2.6)	0.010	3 (0.6)	8 (3.6)	0.003
90-day mRS (IQR)	0 (0–1)	2 (1–4)	<0.001	1 (0–2)	2 (1–4)	<0.001

DNT, door-to-needle time; ENI, early neurological improvement; HT, hemorrhage transformation; sICH, symptomatic intracranial hemorrhage; IQR, interquartile range; LVO, large vessel occlusion; NIHSS, National Institutes of Health Stroke Scale.

**FIGURE 2:**
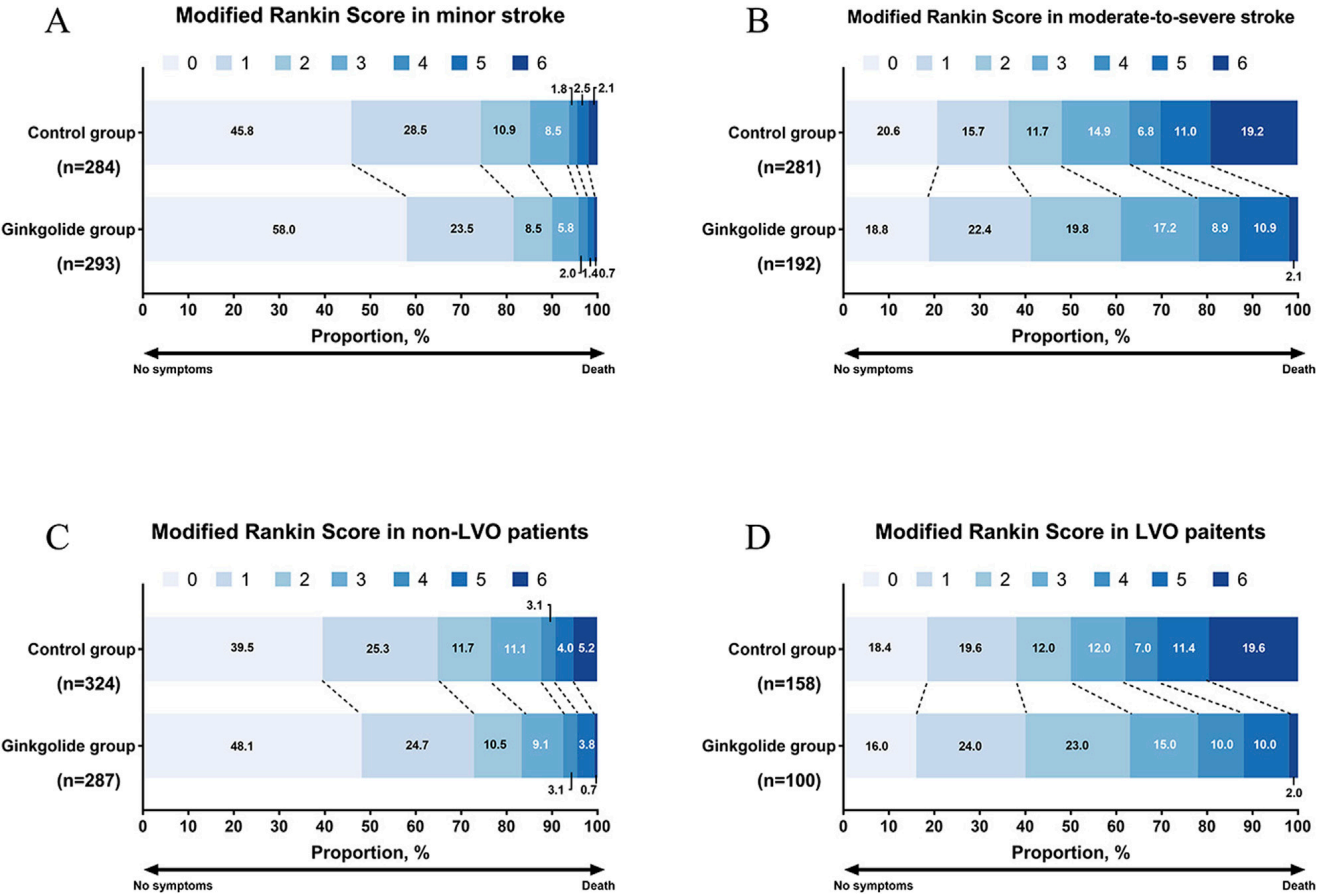
Distribution of modified Rankin scores at 90 days. **(A)** mRS distribution in patients with minor stroke; **(B)** mRS distribution in patients with moderate-to-severe stroke; **(C)** mRS distribution in non-LVO patients; **(D)** mRS distribution in LVO patients.

In LVO patients, patients with a good outcome were younger, had lower baseline NIHSS, lower proportion of CHD and previous stroke, and higher proportion of Ginkgolide^®^ usage. No significant differences in other variables were observed between the two groups. Binary logistic regression analysis showed that, after being adjusted for age, smoking, hypertension, diabetes, previous stroke, and baseline NIHSS, Ginkgolide^®^ usage was independently associated with ENI (OR = 2.621, 95% CI 1.449–4.741, *P* = 0.001) (Table 2). After being adjusted for age, sex, diabetes, CHD, previous stroke, and baseline NIHSS, Ginkgolide^®^ usage was not independently associated with a good outcome (OR = 1.546, 95% CI 0.850–2.811, *P* = 0.154). Ordinal logistic regression indicated that Ginkgolide^®^ usage was not correlated with a favorable shift toward a lower 90-day mRS score (OR = 0.763, 95% CI 0.482–1.209, *P* = 0.249) (Table 2). Figure 2 shows the distribution of mRS values at 90 days.

**TABLE 2:** Multivariable analysis for primary and secondary outcomes in patients with and without LVO.

	Non-LVO					LVO				
	Control	Ginkgolide	Unadjusted *P*	OR (95% CI)	Adjusted *P*	Control	Ginkgolide	Unadjusted *P*	OR (95% CI)	Adjusted *P*
Primary outcome										
90-day good outcome^a^	248/324 (76.5)	239/287 (83.3)	0.039	1.309 (0.822–2.087)	0.257	79/158 (50.0)	63/100 (63.0)	0.041	1.546 (0.850–2.811)	0.154
Secondary outcome										
ENI^b^	253/344 (73.5)	226/300 (75.3)	0.604	1.239 (0.849–1.809)	0.267	96/164 (58.5)	74/101 (73.3)	0.015	2.621 (1.449–4.741)	0.001
90-day ordinal mRS	1 (0–2)	1 (0–2)	0.010	0.670 (0.496–0.905)	0.009	3 (1–5)	2 (1–3)	0.042	0.763 (0.482–1.209)	0.249
Safety outcome										
7d HT^c^	9/249 (3.6)	4/236 (1.7)	0.191	0.477 (0.143–1.590)	0.228	29/128 (22.7)	19/92 (20.7)	0.723	0.985 (0.504–1.925)	0.964
7d sICH^c^	3/249 (1.2)	0/236 (0)	0.091	0.000	0.995	8/128 (6.3)	0/92 (0)	0.015	0.000	0.996

o ^a^Adjusted for age, sex, diabetes, CHD, previous stroke, ginkgolide usage, and baseline NIHSS.

o ^b^Adjusted for age, smoking, hypertension, diabetes, previous stroke, ginkgolide usage, and baseline NIHSS.

f ^c^Adjusted for age, baseline NIHSS, and ginkgolide usage.

OR, odds ratio; CI, confidence interval; mRS, modified Rankin Score; ENI, early neurological improvement; HT, hemorrhagic transformation; sICH, symptomatic intracranial hemorrhage.

In patients with non-LVO, binary logistic regression analysis showed that Ginkgolide^®^ usage was not independently associated with a good outcome (OR = 1.309, 95% CI 0.822–2.087, *P* = 0.257) or ENI (OR = 1.239, 95% CI 0.849–1.809, *P* = 0.267). Ordinal logistic regression indicated that Ginkgolide^®^ usage was correlated with a favorable shift toward a lower 90-day mRS score (OR = 0.670, 95% CI 0.496–0.905, *P* = 0.009).

### Comparison between patients with minor stroke and moderate-to-severe stroke

Table 1 summarized the baseline characteristics in patients with minor stroke and moderate-to-severe stroke. Compared with patients that had a minor stroke, patients in the moderate-to-severe stroke group were older; had a higher DNT and 90-day mRS score (2 (1–4) vs. 0 (0–1), *P* < 0.001), higher proportion of female, LVO, history of AF and CHD, HT, and sICH; and a lower rate of diabetes and Ginkgolide^®^ usage (41.5% vs. 49.9%, *P* = 0.005).

In patients with moderate-to-severe stroke, patients with good outcome were younger; had a lower percentage of females, AF, diabetes, and CHD; and a higher percentage of Ginkgolide^®^ usage (60.9% vs. 48.0%). No significant differences in other variables were observed between the two groups. Binary logistic regression analysis showed that, after being adjusted for age, sex, AF, smoking, diabetes, and CHD, Ginkgolide^®^ usage was independently associated with a good outcome (OR = 1.732, 95%CI 1.146–2.616, *P* = 0.009). After being adjusted for age, sex, smoking, and diabetes, Ginkgolide^®^ usage was independently associated with ENI (OR = 1.607, 95% CI 1.052–2.454, *P* = 0.028). Ordinal logistic regression indicated that Ginkgolide^®^ usage was correlated with a favorable shift toward a lower 90-day mRS score (OR = 0.610, 95% CI 0.438–0.849, *P* = 0.003) (Table 3).

**TABLE 3:** Multivariable analysis for primary and secondary outcomes in patients with different stroke severities.

		Minor stroke					Moderate-to-severe stroke			
	Control	Ginkgolide	Unadjusted *P*	OR (95% CI)	Adjusted *P*	Control	Ginkgolide	Unadjusted *P*	OR (95% CI)	Adjusted *P*
Primary outcome										
90-day good outcome^a^	242/284 (85.2)	264/293 (90.1)	0.074	1.469 (0.859–2.512)	0.160	135/281 (48.0)	117/192 (60.9)	0.006	1.732 (1.146–2.616)	0.009
Secondary outcome										
ENI^b^	199/303 (65.7)	211/298 (70.8)	0.177	1.216 (0.852–1.736)	0.280	206/295 (69.8)	161/205 (78.5)	0.030	1.607 (1.052–2.454)	0.028
90-day ordinal mRS	1 (0–2)	0 (0–1)	0.003	0.610 (0.445–0.837)	0.002	3 (1–5)	2 (1–3)	0.003	0.610 (0.438–0.849)	0.003
Safety outcome										
7d HT^c^	4/226 (1.8)	5/226 (2.2)	0.736	1.249 (0.325–4.795)	0.746	38/215 (17.7)	19/177 (10.7)	0.052	0.576 (0.318–1.045)	0.070
7d sICH^d^	2/226 (0.9)	0/226 (0)	0.156	0.000	0.995	10/215 (4.7)	0/177 (0)	0.004	0.000	0.995

o ^a^Adjusted for age, sex, smoking, AF, diabetes, CHD, and ginkgolide usage.

o ^b^Adjusted for age, female, smoking, diabetes, and ginkgolide usage.

o ^c^Adjusted for age, AF, and ginkgolide usage.

o ^d^Adjusted for age and ginkgolide usage.

OR, odds ratio; CI, confidence interval; mRS, modified Rankin Score; ENI, early neurological improvement; HT, hemorrhagic transformation; sICH, symptomatic intracranial hemorrhage.

In patients with minor stroke, binary logistic regression analysis showed that Ginkgolide^®^ usage was not independently associated with a good outcome (OR = 1.469, 95% CI 0.859–2.512, *P* = 0.160) or ENI (OR = 1.216, 95% CI 0.852–1.736, *P* = 0.280). Ordinal logistic regression indicated that Ginkgolide^®^ usage was correlated with a favorable shift toward a lower 90-day mRS score (OR = 0.610, 95% CI 0.445–0.837, *P* = 0.002) (Table 3).

### Overall characteristics

Multivariable analysis indicated that Ginkgolide^®^ usage was not independently associated with HT in LVO patients (OR = 0.985, 95% CI 0.504–1.925, *P* = 0.964), non-LVO patients (OR = 0.477, 95% CI 0.143–1.590, *P* = 0.228), moderate-to-severe stroke patients (OR = 0.576, 95% CI 0.318–1.045, *P* = 0.070), or minor stroke patients (OR = 1.249, 95% CI 0.325–4.795, *P* = 0.746). Ginkgolide^®^ usage was not associated with sICH in the four subgroups (all *P* > 0.05).

## Discussion

Our subgroup analysis of the GIANT trial found that Ginkgolide^®^ together with IVT in patients with LVO and moderate-to-severe stroke achieved a better outcome than IVT alone. Furthermore, the rate of ENI was higher in the subgroup with LVO. Ginkgolide^®^ combined with thrombolysis did not increase the risk of HT in all the subgroups.

In our study, we proved that Ginkgolide^®^ usage was associated with ENI in patients with LVO, which was in line with previous studies. Pre-clinical studies suggested that ginkgolide improved acute-phase outcomes in both LVO stroke with and without successful reperfusion. In a permanent middle cerebral artery occlusion (MCAO) mouse model, ginkgolide injection reduced the infarction size and improved 5-day neurological outcome, increasing the number and diameter of newly established micro-vessels and cerebral blood flow in the infarcted hemisphere (Zhu et al., 2022). In another transient MCAO (tMCAO) model, ginkgolide B was applied after reperfusion and treated for 3 days, finding that ginkgolide B significantly reduced the infarction volume and enhanced mouse neurological performance at day 7 (Shu et al., 2016). Ginkgolide^®^ was found to be a specific and selective antagonist of the platelet-activating factor (PAF) (Koch, 2005), which is a potent phospholipid mediator that was first described by its ability to cause platelet aggregation and dilation of blood vessels. Suppression of PAF could prevent the activation of platelets in LVO, ameliorating the secondary injury caused by platelet-triggered inflammation. In support of this, the Ginkgolide in Ischemic Stroke patients with large Artery Atherosclerosis (GISAA) trial showed that Ginkgolide^®^ decreased post-stroke PAF and was independently associated with higher NIHSS improvement and 1-month good outcome (mRS 0–2) (Dong et al., 2021). Collectively, these findings explain why Ginkgolide^®^ improves the rate of ENI in patients with LVO receiving IVT.

We proved that Ginkgolide^®^ was associated with extra benefits in patients with moderate-to-severe stroke but not in patients with mild stroke. A previous study by Zhang et al. showed that NIHSS improvement was lower in patients with mild stroke than in those with moderate-to-severe stroke (Zhang et al., 2022). A pre-marketing study including 437 patients found that the benefit of Ginkgolide^®^ was more pronounced in patients with baseline NIHSS >8 (Dong et al., 2020). Similarly, another phase-IV clinical trial investigated the efficacy of Ginkgolide^®^ in a total of 3,652 patients with AIS, showing that patients with NIHSS >8 benefit more from Ginkgolide^®^ than those with NIHSS ≤8 (Dong et al., 2020). Our finding is consistent with previous studies. Generally, moderate-to-severe AIS was usually associated with lower cerebral blood flow, larger baseline infarction volume, and poorer collateral status (Zhang et al., 2010; Lin et al., 2021). By increasing blood flow (Huang et al., 2012) and protecting the blood–brain barrier (Fang et al., 2015), Ginkgolide^®^ may work better in this subgroup.

In our primary result, early Ginkgolide^®^ usage after intravenous rt-PA was not associated with a higher hemorrhagic risk in all patients, further proving that Ginkgolide^®^ was safe in different stroke severities with or without LVO. This result was in accordance with the GISAA trial, which indicated that Ginkgolide^®^ did not increase the risk of hemorrhage in AIS patients with intracranial artery stenosis (ICAS).

Our study had several limitations. First, although a total of 1,113 patients were included in the trial, 18% of the cohort did not perform vessel evaluation on admission, and 24% did not have a follow-up CT scan. Therefore, the credibility of the analysis might be reduced. Second, Ginkgolide^®^ may function through anti-inflammatory pathways, and we did not evaluate the association between serum PAF and other anti-inflammation factors including tumor necrosis factor and interleukin-6. Exploring these indexes may provide further evidence regarding the underlying mechanism of Ginkgolide^®^ in patients with AIS. Third, this is a *post hoc* subgroup analysis, which is prone to generating false-negative results. Finally, we did not include many other factors that are essential in patients with LVO, such as thrombectomy and collateral status, which had a profound impact on the outcomes of patients.

## Conclusion

In summary, Ginkgolide^®^ presented extra benefits in both patients with LVO and moderate-to-severe stroke in the early stage after intravenous rt-PA, suggesting that Ginkgolide^®^ usage is a promising neuroprotective agent in these patients in combination with IVT.

## Data Availability

The raw data supporting the conclusions of this article will be made available by the authors, without undue reservation.
