# Supplementary material for: Efficacy and safety of Ginkgolide with intravenous alteplase thrombolysis in acute ischemic stroke with large vessel occlusion: a subgroup analysis of GIANT
Source: Front Pharmacol. 2024 Aug 30;15:1452174. doi: 10.3389/fphar.2024.1452174 (PMC11392837; doi:10.3389/fphar.2024.1452174)
Supplement: Supplementary file 1 [file Table1.DOCX]

**Supplementary tables**

**Supplementary table I. Univariate comparison of characteristics stratified by intervention.**

|  | Control (n=600) | Ginkgolide^®^ (n=513) | *P* value |
| --- | --- | --- | --- |
| **Demographic data** |  |  |  |
| Age (mean±SD) | 69±13 | 67±12 | 0.076 |
| Female (n, %) | 244 (40.7) | 208 (40.5) | 0.967 |
| **Clinical data** |  |  |  |
| Admission NIHSS (median, IQR) | 5 (3-12) | 5 (2-9) | 0.001 |
| Minor stroke (n, %) | 303 (50.5) | 302 (58.9) | 0.005 |
| DNT (median, IQR) | 47 (38-60) | 42 (34-60) | 0.003 |
| Etiology (n, %) |  |  | <0.001 |
| LAA | 263 (50.9) | 143 (39.8) |  |
| CS | 118 (22.8) | 59 (16.4) |  |
| PAD | 108 (20.9) | 137 (38.2) |  |
| OE | 3 (0.6) | 0 (0) |  |
| UE | 25 (4.8) | 20 (5.6) |  |
| Large vessel occlusion (n, %) | 166 (32.5) | 102 (25.3) | 0.017 |
| Thrombectomy (n, %) | 58 (9.7) | 25 (4.9) | 0.002 |
| ENI (n, %) | 405 (67.7) | 372 (74.0) | 0.024 |
| **Risk factors (n, %)** |  |  |  |
| Smoking | 182 (30.3) | 191 (37.2) | 0.015 |
| Hypertension | 365 (60.8) | 351 (68.4) | 0.008 |
| Coronary heart disease | 48 (8.0) | 48 (9.4) | 0.422 |
| Atrial fibrillation | 126 (21.0) | 83 (16.2) | 0.040 |
| Diabetes | 86 (14.3) | 88 (17.2) | 0.196 |
| Previous stroke | 62 (10.4) | 74 (14.5) | 0.043 |
| **Follow up index** |  |  |  |
| Discharge mRS (median, IQR) | 1 (1-3) | 1 (0-2) | <0.001 |
| 90-day mRS (median, IQR) | 1 (0-3) | 1 (0-2) | <0.001 |
| 90-day good outcome (n, %) | 376 (66.5) | 381 (78.6) | <0.001 |
| 7-day HT (n, %) | 42 (9.5) | 24 (6.0) | 0.054 |
| 7-day sICH (n, %) | 12 (2.7) | 0 (0) | 0.001 |

SD, standard deviation; NIHSS, National Institute of Health stroke scale, IQR, interquartile range; DNT, door-to-needle time; LAA, large artery atherosclerosis; CS, cardiogenic stroke; PAD, penetrating artery disease; OE, other etiology; UE, undetermined etiology; ENI, early neurological improvement; mRS, modified Rankins Score; HT, hemorrhagic transformation; sICH, symptomatic intracranial hemorrhage.
